# High Performance Mixed-Matrix Electrospun Membranes for Ammonium Removal from Wastewaters

**DOI:** 10.3390/membranes11060440

**Published:** 2021-06-11

**Authors:** Shu-Ting Chen, Sumith Ranil Wickramasinghe, Xianghong Qian

**Affiliations:** 1 Department of Chemical Engineering, University of Arkansas, Fayetteville, AR 72701, USA; sc068@uark.edu (S.-T.C.); swickram@uark.edu (S.R.W.); 2 Department of Biomedical Engineering, University of Arkansas, Fayetteville, AR 72701, USA

**Keywords:** ammonium removal, wastewater, mixed-matrix membranes, zeolite, electrospinning

## Abstract

Mixed-matrix electrospun membranes were developed to investigate ammonium removal from low ammonium concentration wastewaters for the first time. Particles derived from the inexpensive zeolite 13X were successfully incorporated into polyethersulfone (PES) matrices. The fabricated mixed-matrix electrospun membranes demonstrate high ammonium removal capacity reaching over 55 mg/g_zeolite_, more than 2.5 times higher than the previously fabricated mixed-matrix membranes via non-solvent induced phase inversion. Moreover, the membranes fabricated exhibit high permeability and ease of regeneration. Over 90% of total ammonium nitrogen (TAN) can be removed from low TAN wastewaters such as aquaculture wastewaters. In addition to zeolite 13X, other zeolite particles including zeolite Y, zeolite 3A and 4A were also incorporated into the membrane matrix. The inexpensive zeolite 13X show the highest ammonium exchange capacity. Particle type, loading and the level of its dispersion all affect TAN removal capacity.

## 1. Introduction

The biological production of ammonia (NH_3_) is part of the natural metabolic process across all fauna, from humans to fish. Ammonia is a major protein metabolite released to the environment. It is also an essential nutrient for plants and a key component of the nitrogen cycle. Depending on the acidity or pH of the aquatic system, the equilibrium between the molecular form (NH_3_) or protonated form (NH_4_^+^) can shift dramatically with one or the other being the dominant species. Environmental protection agency (EPA) regulates the discharging limit of total ammonia nitrogen (TAN) including both NH_4_^+^ and NH_3_ from the wastewater treatment plants. Municipal wastewater treatment typically employs aerobic process to convert TAN to nitrite (NO_2_^−^) and further to nitrate (NO_3_^−^). Nitrate is ultimately converted to N_2_ and released to air via the anaerobic process. The biological conversion process is generally effective once established but slow. However, it does take a long time to establish the bacterial culture during the initial start-up phase. In addition, it may be ineffective when there is a sudden surge of TAN in the wastewater as biological conversion is slow.

Ammonia is released to the aquaculture water during protein metabolism by fish and via bacterial digestion of organic matter. Ammonia is rather toxic to fish and has to be maintained below 0.05 mg/L (ppm). This puts the maximum TAN in aquaculture water to be ~1.5 ppm at neutral pH. In order to maintain this level of TAN, the aquaculture water has to be often partially replaced with fresh water. Zeolite materials have been employed for the removal of ammonium ion from wastewaters [1,2,3,4,5,6]. Previous studies indicate that the inexpensive natural zeolite 13X can perform ion exchange with ammonium ion with high capacity [6]. However, zeolite 13X is not stable in water and cannot be applied effectively as an ammonium absorbent. Our previous work [7] has shown that a mixed-matrix membrane incorporating cheap natural zeolite 13X particles can effectively remove NH_4_^+^ from aquaculture wastewater via ion-exchange to reach TAN concentration below 1 ppm. No leach of zeolite materials has been observed. Three different methods were used to fabricate the mixed-matrix membranes and fibers. One involves non-solvent induced phase inversion by mixing the zeolite particles with the polysulfone (PSU) in 1-methyl-2-pyrrolidinone (NMP). The maximum amount of zeolite incorporated without compromising the mechanical property of the membrane is 50% of the PSU amount. Our results indicate that the higher the amount of zeolite particles incorporated, the higher the capacity of the mixed-matrix membrane for removing ammonium ion via ion-exchange. The maximum capacity reached is 19.8 mg of TAN per g of zeolite (mg/g_zeolite_) for the membrane with 15% PSU and zeolite loading of 50% PSU. The second approach is the formation of zeolite-containing fibers by mixing the zeolite with PSU in NMP similar to the preparation of mixed-matrix membrane. The prepared solution was then dispensed into water via a syringe. These fibers exhibit a maximum ammonium exchange capacity of 5.4 mg/g_zeolite_ at 15% PSU and zeolite loading of 50% PSU. The third approach involves the pore-filling of zeolite through the supporting structure from the back of an existing 30 kD polyethersulfone (PES) membrane. The maximum capacity reached is 10.4 mg/g_zeolite_. In all these approaches, it seems that the capacity is limited by the zeolite loading.

In order to incorporate more zeolite particles into the membrane matrix, electrospinning technique has been used here to fabricate mixed-matrix membranes. Fabrication of electrospun membranes involve the application of a high voltage to produce nanofibers from a charged polymer solution or melt. The surface topology, fiber morphology, and orientation of electrospun membranes depend strongly on the nature of the casting solution and the operating condition. The rheology of the polymer solution is most critical to the structure and properties of the spun nanofiber. Electrospun fibers are also affected by the voltage applied, solution flow rate, and tip-collector distance, as well as chamber temperature and humidity. Careful control of the operating condition and casting solution could lead to the production of smooth, defect-free, highly porous non-woven nanofiber membranes. In contrast to phase inversion method, the electrospun membranes have relative uniform pore sizes with high pore interconnectivity and high porosity (~80%) [8,9]. In addition, electrospun membranes have robust mechanical properties and the possibility to incorporate different additives, such as zeolite particles. These properties lead to the wide industrial application of electrospinning for membrane fabrications [10,11]. Besides membranes used for separation, electrospun materials have significant potential for applications in bioengineering, catalysis, and many other areas [12,13,14,15,16]. Electrospun membranes have been extensively studied for biomedical applications due to their biocompatibility and biodegradability [17]. Previously we fabricated weak anion exchange electrospun membranes for protein purifications [18]. Here mixed-matrix electrospun membranes are synthesized for ammonium removal from wastewaters containing both low and high TAN concentration levels.

## 2. Materials and Methods

### 2.1. Materials

Polyethersulfone (PES; MW ~60,000) in powder form as membrane material was purchased from BASF (Ludwigshafen, Germany). *N*, *N*-dimethylformamide (DMF) (≥99.8% ACS), sodium hypochlorite (12.5% in aqueous solution) and sodium chloride (NaCl) (ACS grade) were purchased from VWR (Radnor, PA, USA). Ammonium chloride (≥99%) was purchased from Alfa Aesar (Haverhill, MA, USA). Liquid phenol (>89%) was purchased from Sigma–Aldrich (St. Louis, MO, USA). Sodium nitroprusside dihydrate (≥98%) was purchased from MP biomedicals (FKA ICN BIOMED) (Santa Ana, CA, USA). Sodium hydroxide (≥98%, ACS grade) was purchased from Amresco (Solon, OH, USA). Sodium citrate dehydrate (≥99%) was purchased from J.T. Baker (Phillipsburg, NJ, USA). All chemicals were used without further purifications. Zeolite 13X molecular sieve (Stream Chemicals, 1/16” pellets (Linde 13X)), zeolite Y, sodium molecular sieve 4A powder, and molecular sieve 3A powder were purchased from Alfa Aesar (Haverhill, MA, USA). Deionized water (DI) was obtained from Milli-Q ultrapure water purification system (Millipore, Billerica, MA, USA). A commercial PAN membrane, (MWCO 400,000, Ultura) which was used as a support layer for the electrospun nanofibers, was provided by Ultura (Oceanside, CA, USA).

### 2.2. Fabrication of Mixed-Matrix Nanofibrous Membranes

Zeolite 13X particles were grinded mechanically to obtain fine a powder. After grinding, particle size of 13X was measured using laser diffraction. The distribution of grinded particles was shown previously [7]. The particle size ranges from 0.6 to 6 μm. with an average diameter of ~1.5 μm. An electrospinning process was used to fabricate PES nanofibers. Zeolite 13X and PES powders with a fixed weight ratio were dissolved in DMF under stirring at 70 °C. Thereafter, a homogeneous casting solution was obtained after 24 h of stirring and mixing. The solution was then injected into a syringe pump.

Nanofibers were deposited onto the backside of the PAN membrane during the electrospinning process. Here the syringe pump flow rate was fixed at 0.5 mL h^−1^. The applied voltage was kept at 25 kV and the distance between the needle tip and fiber collector was maintained at 25 cm. These are the critical parameters to control the fiber diameter and the properties of the fibrous membranes formed. The *x*%PES–*y*%zeolite was used to denote the membranes with different compositions, where *x* represents the wt% of PES to the polymer-solvent mixed casting solution, *y* represents the wt% of 13X with respect to PES in the solvent mixture.

### 2.3. Characterization

#### Membrane Morphology

The membrane surface and cross-section morphology were investigated by field-emission scanning electron microscopy FESEM Model S-4800 (Hitachi Co., Tokyo, Japan) with 1 kV electron beam. The membrane samples were dried in vacuum oven at 40 °C at least 24 h to remove residue solvent and moisty before sample preparation. The membrane was then dipped in liquid nitrogen for a few seconds to obtain a uniform cross-section. Finally, the membrane was cut into pieces with appropriate size and subsequently mounted on the SEM sample holder by carbon conductive tabs (Ted Pella, Redding, CA, USA) for imaging.

### 2.4. Performance of Mixed-Matrix Membranes

Filtration experiments were performed using a stirred normal filtration cell (Sterlitech corporation, Kent, WA, USA) connected to a pressurized feed vessel from a nitrogen tank. During filtration, the feed solution was stirred to minimize concentration polarization. Prior to each test, the membrane coupons were compacted by filtering DI water until a steady permeate flux was obtained. The permeate flux was calculated based on the time-derivative of the permeate mass, which was measured by collecting the filtered water on a digital balance (Mettler Toledo, PL602-s) connected to a computer.

To evaluate membrane performance, 7 to 60 ppm TAN feed solutions containing ammonium chloride (NH_4_Cl) were filtered through the compacted membranes using the dead-end filtration cell. Filtration pressure was adjusted for testing different membranes in order to control the permeate flux and subsequently the residence time for ammonium ion exchange. Multiple permeate samples were collected for each membrane to determine its ammonium removal capacity. TAN concentration in the sample was determined by the Phenol Hypochlorite Method [19] using a UV–VIS spectrophotometer (Thermo Scientific™ GENESYS 10S UV-Vis, Waltham, MA, USA). TAN removal rate was calculated by the concentration of TAN in the filtrate collected divided by the concentration of TAN in the feed using the following equation:

(1) $$TAN\;removal\;\%=\frac{C_{permeate}}{C_{feed}}\times 100$$

where $C_{filtrate}$ and $C_{feed}$ represent the concentration of TAN in the permeate and feed solutions respectively. TAN removal capacity was determined by calculating the total amount of TAN removed over the course of filtration experiment divided by the amount of zeolite incorporated in the membrane in mg/g using the following equation:

(2) $$TAN\;Removal\;Capacity=\frac{\sum C_{permeate}\times V_{permeate}}{W_{zeolite\;13X}}$$

## 3. Results and Discussion

### 3.1. The Effects of Zeolite Loading on TAN Removal

Our previous work [7] shows that ammonium uptake by zeolite 13X particles in the solution follows the Freundlich isotherm with a multilayer inhomogeneous adsorption mechanism. The adsorption capacity coefficient K_F_ is about 17.6 mg/g zeolite. Moreover, the adsorption of ammonium ions in the solution is rather rapid reaching equilibrium with less than 30 min. The adsorption kinetics follows the pseudo second order. The equilibrium NH_4_^+^ adsorption capacity was calculated to be 28.5 mg TAN per gram of zeolite. However, adsorption capacity is strongly dependent on the experimental condition including the ratio of zeolite to TAN.

To investigate the effects of zeolite 13X loading on ammonium removal, mixed-matrix membranes were fabricated incorporating zeolite 13X equivalent to 0, 50 and 100 wt% with respect to PES amount in the casting solution. The filtration experiments were conducted using 45 millimeter (mm) in diameter cut membrane coupons from the fabricated electrospun nanofiber membranes. Prior to ammonium exchange experiments, membranes were compacted using DI water until a stable flux was obtained. Since the mechanism for ammonium removal from the feed solution is via ion-exchange process, residence time of the feed solution with the membrane is an important parameter. Based on the DI water fluxes of the fabricated mixed-matrix membranes and our earlier studies [7], a flux of about 70 LMH can ensure sufficient time for the ion-exchange process while maintaining the efficiency of the filtration process. More details on the effects of flux on ammonium removal will be discussed in the next section.

**Figure 1:**
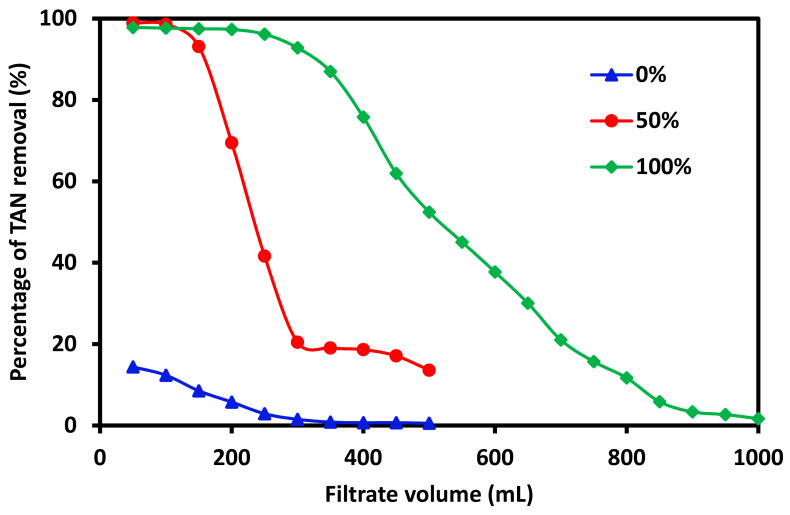
Percentage of TAN removed as a function of filtrate volume collected for three mixed matrix membranes incorporating 0, 50, and 100 wt% zeolite 13X with respect to PES polymer in the performance of membrane with various zeolite loadings.

Here, a flux of 70 LMH was maintained by adjusting the pressure for all three membranes with different zeolite 13X loadings. Figure 1 shows the percentage of TAN removed as a function of the filtrate volume collected for three membranes. All feed solutions contain 15 ppm TAN. Permeate samples were analyzed for each 50 mL collected. The 20%PES-0%Zeolite13X membrane without incorporating zeolite13X showed less than 20% TAN removal during the first 200 mL of filtrate collected. Thereafter, TAN removal quickly decreases to almost 0 for the remaining 300 mL of filtrate collected. This indicates that PES membranes possess some negatively charged sites and could perform limited ammonium ion-exchange with the feed solution. However, its TAN removal capacity is very limited. When 50 wt% of zeolite 13X was incorporated into the membrane (20%PES-50%Zeolite13X), the membrane showed over 97% TAN removal during the first 100 mL of filtrate. However, TAN removal rate quickly decreased during the next 200 mL of filtrate to just over 20%. The TAN removal rate continued to decrease during the remaining 200 mL of filtrate. However, when 100 wt% of zeolite 13X was incorporated into the membrane (20%PES-100%Zeolite13X), TAN removal rate consistently reached over 95% for the first 250 mL permeate collected. Thereafter, TAN removal rate decreased to about 93% at 300 mL filtrate and ~52% at 500 mL reaching ~12% at 800 mL filtrate. It can be seen that the amount of TAN removed is directly proportional to the amount of zeolite 13X incorporated into the membrane in agreement with our previous findings [7].

**Figure 2:**
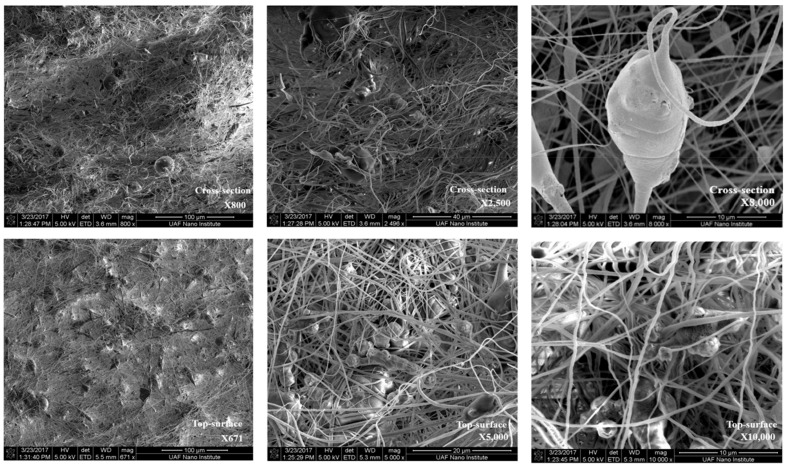
SEM images of cross-sectional surfaces (**top** panel) and top surfaces (**bottom** panel) of fabricated mixed matrix membrane (20%PES-100%Zeolite13X) with increased left to right magnifications.

**Figure 3:**
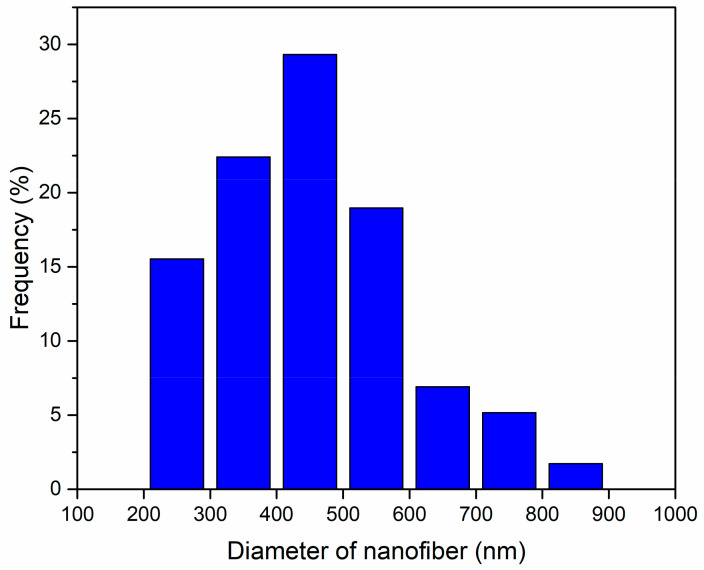
The nanofiber diameter distribution of electrospun 20%PES-100%Zeolite13X membranes based on the images from SEM.

Figure 2 shows the SEM images of the cross-sectional surfaces (top panel) and membrane top surfaces (bottom panel) for one of the fabricated mixed-matrix membranes at 20%PES-100%Zeolite13X composition. The magnification increases from left to right for the images taken. The zeolite particles can be visibly seen. Fiber diameters have also been measured based on these images as shown in Figure 3. Electrospun nanofiber diameters varied from 200 to 900 nm with the highest percentage appeared between 400 and 500 nm. The diameters were measured without taking zeolite particles into consideration since these particles range from 0.6 to 6 μm, with an average diameter of 1.5 μm.

### 3.2. The Effects of Filtration Flux on Ammonium Exchange and Removal

**Figure 4:**
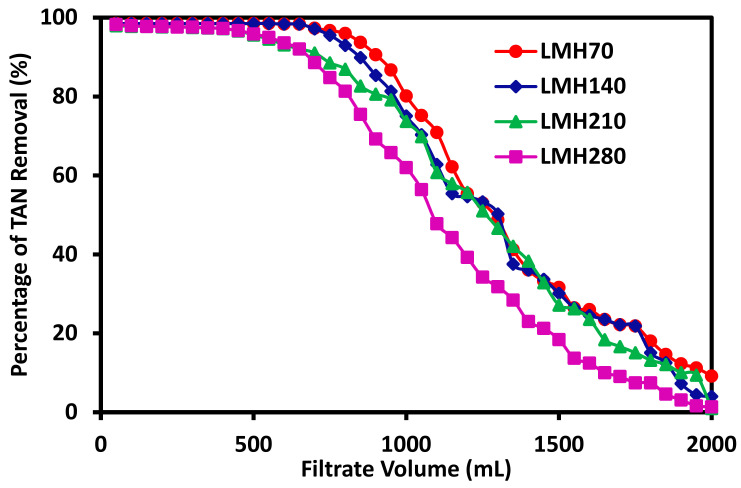
Variation of TAN removal rate with filtration flux with a 2000 mL feed containing 6 ppm TAN for the fabricated membranes with the same composition but slightly different weight as shown in Table 1.

Since the mechanism for ammonium ion removal from the wastewaters is ion-exchange, both the kinetics and thermodynamics of the ion-exchange process are important and need to be studied. Here, electrospun mixed-matrix membranes with the same composition of 20%PES-100%Zeolite13X were fabricated with approximately the same weight as shown in Table 1. These membranes were tested with 6 ppm TAN synthetic wastewaters at a flux of 70, 140, 210, and 280 LMH, respectively. The percentage of TAN removed in every 50 mL filtrate was measured and plotted as a function of the feed volume, as shown in Figure 4. Different flux values represent different kinetics for the ammonium ion exchange process. A total of 2000 mL feed volume was tested. It can be seen that for ammonium removal rate reached over 90% during the initial 650–850 mL of the feed depending on the flux. Thereafter, the flux declined rapidly over the next 1000 mL of the feed and eventually reduced to less than 10% at the last 50 mL of the filtrate collected. However, there are some differences between experiments with different fluxes. The total amount of TAN removed for each experiment was calculated and shown in Table 1. Since the actual membrane weight thereby its zeolite 13X content is slightly different, the total TAN amount removed does not necessarily reflect the effect of flux on the TAN removal capacity. The TAN removal capacity in mg over the total amount of zeolite 13X incorporated in the membrane in g was shown in the last column in Table 1. It can be seen that the lowest flux at 70 LMH exhibited the highest TAN removal capacity reaching 53.7 mg/g. The removal capacity reduced slightly to ~52.2–52.4 mg/g for the 140 and 210 LMH experiments. The TAN removal capacity reduced much more to 48.8 mg/g for filtration experiment at 280 LMH flux. For the remaining experiments, a filtration flux of 70 LMH was kept. It is surprising to observe that the ammonium removal capacity for the electrospun membranes incorporating zeolite 13X is almost twice as high as the capacity of zeolite 13X particles without being incorporated. This is likely due to the aggregation of the naked particles when they were immersed in the solution. On the other hand, the zeolite particles in the membranes are dispersed and exposed to the feed containing the ammonium ions leading to the significant increase in the ammonium exchange capacity.

### 3.3. The Effects of TAN Concentration on Its Removal Capacity

**Figure 5:**
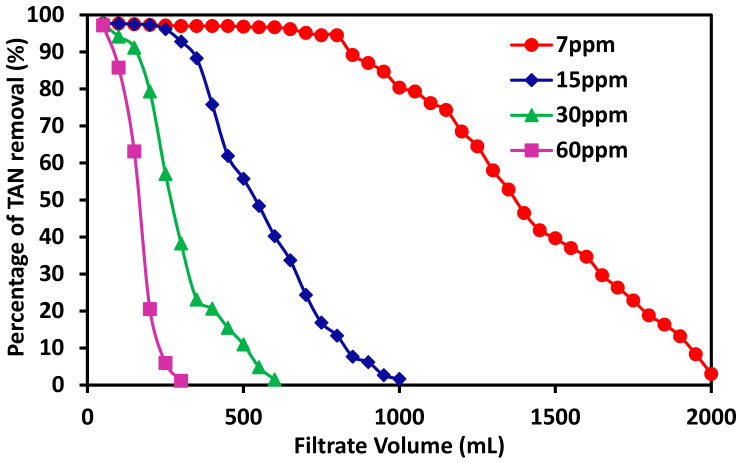
The effects of TAN concentration in the feed on its removal rate at LMH 70 for the fabricated membranes with the same composition.

The effects of feed concentration on the dynamic exchange capacity were also investigated. The feed solutions containing 7, 15, 30, and 60 ppm TAN concentrations were filtered through freshly fabricated membranes with 20%PES-100%Zeolite13X with similar weights. The TAN removal rate was plotted as a function of filtration feed volume passed through the membrane as shown in Figure 5. The flux was kept at 70 LMH based on previous studies. It can be seen that TAN removal rate reduced substantially as the TAN concentration in the feed multiplies. Table 2 lists the masses of the membranes fabricated, the corresponding feed concentrations, the total amount of TAN removed, and TAN removal capacity in mg of TAN per gram of zeolite 13X incorporated in the membrane. It can be seen that the dynamic exchange capacity does not change much when the TAN concentration increases. The ammonium ion exchange seems to be rather rapid and is not affected by the high amount of TAN present in the feed. This is consistent with the previous flux data that TAN removal is a rather rapid process and that only a slight decrease in the capacity was observed even at the highest filtration flux of 280 LMH. This also indicates the potential application of these MMMs for the removal of TAN at low concentration feed streams, such as aquaculture wastewaters, but also for other industrial processes producing high TAN feed streams.

### 3.4. The Effects of Membrane Regeneration on Its Performance

**Figure 6:**
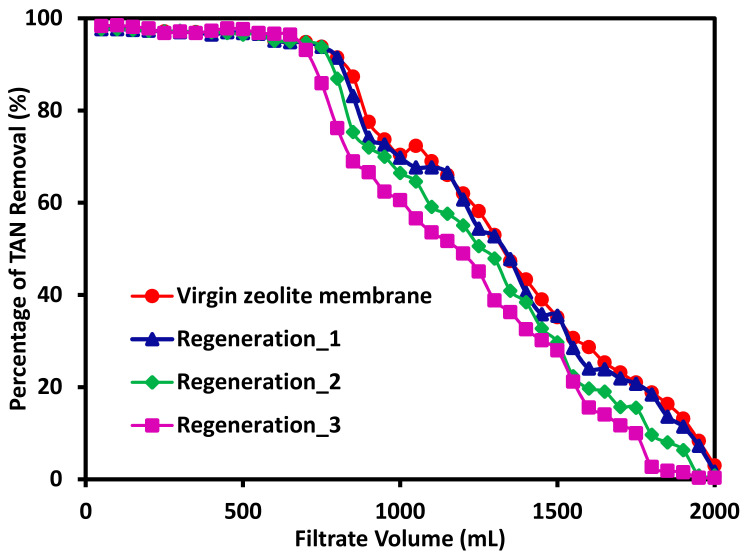
The TAN removal rates as a function of feed volume with the fabricated 20%PES-100%Zeolite13X mixed-matrix membranes over multiple removal and regeneration cycles.

The previous studies on the effects of filtration flux and feed concentration on the TAN removal rate and TAN removal capacity for the fabricated mixed-matrix membranes used freshly made membranes with the same composition and approximately the same weight. The reuse and regeneration of the membranes were also investigated. Here, after the membrane was filtered with a 2000 mL feed solution containing 6 ppm TAN, the regeneration of the membranes was conducted by filtering the membrane using 2 M NaCl solution at 70 LMH until TAN in the filtrate cannot be detected. This process takes about 12 h and 2000 mL of the salt solution. Since NH_4_^+^ ion and Na^+^ have the same charge and are very similar to each other in terms of their ionic radii, it is expected that the exchange between the two ions are rather efficient. Indeed, it appears that after the first regeneration of the membrane, the TAN removal rate remains more or less the same as shown in Figure 6. The filtrate volumes with 90% TAN removal for both the virgin membrane and the regenerated membrane reached 850 mL. However, there is some slightly loss of TAN removal capacity. During the subsequent 2nd and 3rd regenerations, only 750 mL and 700 mL, respectively, of the filtrates reached 90% TAN removal rate. The loss of membrane capacity for TAN removal is more apparent as also can be seen clearly in Figure 6. This loss of capacity is likely due to the diffusion of the NH_4_^+^ ions into the inner regions of the zeolite 13X particles leading to the unavailability of those sites for further adsorption of the ammonium ions. However, it can be seen from this study that these mixed-matrix membranes can be potentially used multiple times after regeneration using a 2 M NaCl salt solution. Moreover, if NaNO_3_ salt solution is used instead of NaCl, NH_4_^+^ can be recovered in the format of NH_4_NO_3_ solution, which can be directly used as a fertilizer. Not only these mixed-matrix membranes can be employed to remove TAN, but also N nutrient can be easily recovered by another ion-exchange process. Further studies are underway to investigate nutrient recovery.

### 3.5. The Effects of Zeolite Type on the Performance of TAN Removal

**Figure 7:**
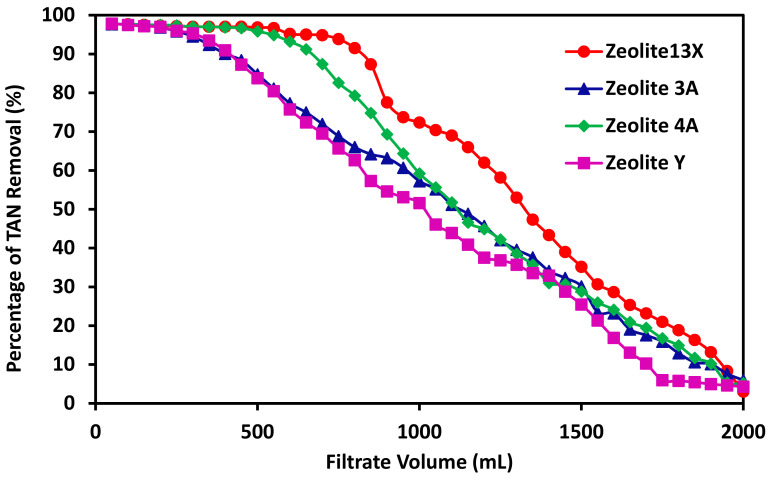
The percentage of TAN removal as a function of the filtrate collected for mixed matrix membranes incorporating different zeolite particles of 13X, 3A, 4A, and Y. The membranes have the same mass composition of 20%PES-100% Zeolite.

Mentioned previously, zeolite 13X is a rather inexpensive and abundant natural zeolite from volcanic ashes, it will not contribute much to the cost of fabricating these mixed-matrix membranes. On the other hand, there are many other zeolite types available including zeolite 3A, 4A, and Y. These zeolite particles possess specific properties such as high purity and with specific pore sizes. They also have a different Al/Si ratio which is an important indicator for the ion-exchange capacity of the zeolites. Figure 7 shows the percentage of TAN removed as a function of the filtrate volume collected for the mixed-matrix membranes incorporating zeolite 13X, 3A, 4A, and Y. All the membranes contain 20%PES and the same weight percentage of the zeolite particles. It can be seen that zeolite 13X demonstrates the highest TAN removal efficiency, followed by zeolite 4A, zeolite 3A with zeolite Y possessing the lowest TAN removal efficiency.

Since these membranes incorporating different zeolite particle types have slightly different weights, the TAN removal capacity was calculated, as shown in Table 3. The weight of the membrane, the flux value for the filtration experiments, the permeate volume reached 90% TAN removal and the total amount of TAN removed are also listed in Table 3. It can be seen that zeolite 13X has the highest TAN removal capacity at 52.5 mg/g. Zeolite 4A has the second highest TAN removal capacity at 48.1 mg/g. The other two zeolite materials 3A and Y have substantially reduced capacity at 43.1 and 43.6 mg/g, respectively.

The differences between these different zeolite types can be due to several reasons. One of the major differences between these zeolite types is their ion-exchange capacity. Ion-exchange capacity is strongly dependent on the Si/Al ratio for the zeolite material. The higher the Si/Al ratio is, the lower the ion-exchange capacity will be. The higher Al content increases the negative charge density of the zeolite. For both zeolite 4A and 3A, the Si/Al ratio is 1. For zeolite Y, the ratio is 2.46. For zeolite 13X is 1.23. Clearly zeolite 4A and 3A should have the highest ion-exchange capacity. However, pore size of the zeolite cages also plays an important role, zeolite 3A, 4A, and 13X have pore sizes of 3, 4, and 10 Å, respectively. Smaller pore size tends to limit the passage of the hydrated ions and their diffusion to the binding sites. As a result, zeolite 13X has the highest ammonium ion exchange capacity, zeolite Y and 3A have similarly low ammonium ion exchange capacity. Moreover, the filtrate volume with more than 90% TAN removal for each membrane is also shown in Table 3. The highest volume is zeolite 13X with 850 mL, followed by zeolite 4A at 650 mL with zeolite 3A and Y at 400 mL consistent with the capacity values. In addition to the ammonium exchange capacity, cost of these zeolites is also a critical consideration of these mixed- matrix membrane with regard to applications for ammonium removal. Zeolite 4A, 3A, and Y are significantly more expensive than zeolite 13X. Mixed-matrix membranes incorporating zeolite 13X appear to be the best performing membranes for ammonium removal applications.

### 3.6. Comparison with Other Zeolite Mixed-Matrix Membranes

The performances of electrospun mixed-matrix membranes and previously fabricated membranes via phase inversion or pore filling on TAN removal are compared and listed in Table 4. The 13X pore-filled PES membrane (MWCO = 30 kDa, EMD Millipore) has the lowest TAN removal capacity at 10.4 mg/g_zeolite_. The phase inversion mixed-matrix membrane exhibits an intermediate TAN removal capacity at 19.8 mg/g_zeolite_. The electrospun membrane has the highest capacity of 55.1 mg/g_zeolite_. The low capacity for pore-filled membrane is clear since zeolite particles are aggregated together when filled in the pores of the PES ultrafiltration membrane as discussed previously. For the membrane fabricated via phase inversion, zeolite particles are more dispersed in the polymer matrix compared to pore-filled membrane leading to an increase in the ion exchange capacity. For the electrospun membrane, it is more porous compared to the corresponding phase inversion one leading to a significant increase in the ion-exchange capacity by almost 3 times. Besides a significantly higher capacity, the loading of zeolite in the electrospun mixed-matrix membrane is also much higher leading to its superior performance.

Table 4 shows the capacity in terms of the amount of water can be treated per membrane area in L/m^2^ if 90% of the TAN is removed in the filtrate assuming that the concentration of TAN in the wastewater is 7 ppm. It can be seen that phase inversion fabricated membrane has the lowest volume TAN removal capacity. This is due to the limited loading of zeolite 13X particles possible for the mixed-matrix membrane without compromising its mechanical integrity. The pore-filled membrane has a higher capacity due to the fact that more zeolite particles can be filled and incorporated into the membrane pores. The electrospun membrane has the highest volume capacity at 750 L/m^2^. This is due to the fact that electrospun membrane is able to incorporate significantly more zeolite particles and that the membrane is more porous compared to the corresponding phase inversion membrane. Electrospun mixed-matrix membrane can treat over 21 times more wastewater than phase inversion counterpart.

## 4. Conclusions

Electrospun mixed-matrix membranes incorporating natural zeolite particles 13X were fabricated for the removal of ammonium ions from the wastewaters. These fabricated membranes demonstrate a high ion exchange capacity and high permeability. The capacity for removal of TAN reaches 55 mg/g_zeolite_ incorporated, over 2.5 times higher than the previously fabricated mixed-matrix membranes via phase inversion. The capacity is not affected by the flux in the range of 70 to 210 LMH due to the rapid ion exchange process. However, when the flux increased to 280 LMH, a light decrease in exchange capacity was observed. In addition, the TAN removal capacity remains the same when the TAN concentration in the feed increases from 7 to 60 ppm. The fabricated electrospun membrane has a throughput of 750 L/m^2^ to reduce the synthetic 7 ppm TAN feed to less than 1 ppm. The membranes are stable and can be regenerated multiple times by filtering 2 M NaCl through the membrane. No leaching of zeolite particles has been observed. Compared to zeolite Y, 4A, and 3A particles, mixed-matrix electrospun membranes incorporating the naturally occurring inexpensive zeolite 13X exhibits the highest ammonium exchange capacity.

## Figures and Tables

**Table 1:** The TAN removal capacity as a function of filtration flux.

Membrane Composition	Membrane Weight (g)	Flux (LMH)	Total TAN Removed (mg)	TAN Removal Capacity (mg/g_zeolite_)
20%PES-100%Zeolite13X	1.18 ± 0.06	70	31.7 ± 2.26	53.7 ± 3.83
20%PES-100%Zeolite13X	1.18 ± 0.09	140	30.8 ± 1.02	52.2 ± 1.73
20%PES-100%Zeolite13X	1.10 ± 0.09	210	28.8 ± 1.89	52.4 ± 3.44
20%PES-100%Zeolite13X	1.05 ± 0.10	280	25.6 ± 2.53	48.8 ± 4.87

**Table 2:** TAN removal capacities of fabricated mixed-matrix membranes with the composition of 20%PES-100%Zeolite13X at feed concentrations of 7, 15, 30, and 60 ppm, respectively.

Membrane Composition	Membrane Weight (g)	Flux (LMH)	Feed Conc. (ppm)	Total TAN Removed (mg)	TAN Removal Capacity (mg/g_zeolite_)
20%PES-100%Zeolite13X	1.15 ± 0.09	70	7	31.7 ± 1.38	55.1 ± 2.38
20%PES-100%Zeolite13X	1.16 ± 0.07	70	15	31.5 ± 1.52	54.4 ± 2.62
20%PES-100%Zeolite13X	1.20 ± 0.13	70	30	31.1 ± 2.17	51.8 ± 3.62
20%PES-100%Zeolite13X	1.17 ± 0.11	70	60	31.4 ± 2.01	53.7 ± 3.44

**Table 3:** TAN removal capacities for mixed-matrix membranes incorporating different zeolite types with the same mass composition and the feed concentration of 6 ppm. The weight of the membrane, and the filtrate volume with more than 90% TAN removal, the total amount of TAN removed are also shown.

Membrane Composition	Membrane Weight (g)	Flux (LMH)	Permeate Volume with >90% TAN Removal (mL)	Total TAN Removed (mg)	TAN Removal Capacity (mg/g_zeolite_)
20%PES-100%Zeolite 13X	1.15 ± 0.07	70	800	30.2 ± 0.75	52.5 ± 1.30
20%PES-100%Zeolite Y	1.08 ± 0.11	70	400	23.6 ± 1.87	43.6 ± 3.46
20%PES-100%Zeolite 3A	1.12 ± 0.09	70	400	24.2 ± 1.79	43.1 ± 3.19
20%PES-100%Zeolite 4A	1.13 ± 0.08	70	650	27.2 ± 1.59	48.1 ± 2.81

**Table 4:** TAN removal capacity comparison among three different mixed-matrix membranes.

Composite Membrane	TAN Removal Capacity (mg/g_zeolite_)	TAN Removal Volume Capacity * (L/m^2^)
Phase Inversion Mixed-Matrix Membrane (15% PSU-50% Zeolite 13x)	19.8	35
Pored-Filled	10.4	140
Electrospun Mixed-Matrix Membrane (20% PES-100% Zeolite 13x)	55.1	750

* The volume of the wastewater treated is based on the water contains 7 ppm TAN only without any other competitive ions present.

## Data Availability

Data available upon request.
